# FEZF1-AS1在非小细胞肺癌中的研究进展

**DOI:** 10.3779/j.issn.1009-3419.2020.101.05

**Published:** 2020-04-20

**Authors:** 槿 陈, 荣 尹, 歆茵 刘

**Affiliations:** 1 211166 南京，南京医科大学 Nanjing Medical University, Nanjing 211166, China; 2 210009 南京，江苏省肿瘤防治研究所/南京医科大学附属肿瘤医院 Jiangsu Institute of Cancer Research, Nanjing Medical University Affiliated Cancer Hospital, Nanjing 210009, China; 3 210029 南京，南京医科大学第一附属医院肿瘤科 Department of Oncology, First Affiliated Hospital of Nanjing Medical University, Nanjing 210029, China

**Keywords:** FEZF1-AS1, 长非编码RNA, 肺肿瘤, FEZF1-AS1, LncRNA, Lung neoplasms

## Abstract

现如今越来越多的证据表明，长非编码RNA（long non-coding RNA, lncRNA）在肿瘤发生及发展中起着重要的作用。作为一种新发现的lncRNA，FEZ家族锌指1反义RNA1（FEZ family zinc finger 1-antisense RNA 1, FEZF1-AS1）在非小细胞肺癌（non-small cell lung cancer, NSCLC）等恶性肿瘤中表达上调，且FEZF1-AS1的异常表达与NSCLC患者的临床特征及预后有关。此外，FEZF1-AS1可以通过多种机制调节肺癌细胞增殖、迁移和侵袭等生物学过程，可能是潜在的NSCLC治疗新靶点。本文就FEZF1-AS1在NSCLC中的最新研究进展进行阐述。

## Abstract (en)

Nowadays, accumulating evidence indicates that long non-coding RNA (lncRNA) play vital roles in tumorigenesis. As a newly discovered lncRNA, FEZ family zinc finger 1-antisense RNA 1 (FEZF1-AS1) is markedly upregulated in various malignant tumors including non-small cell lung cancer (NSCLC). Aberrant expression of FEZF1-AS1 is related to clinical characteristics of patients with NSCLC and suggests poor prognosis. Moreover, FEZF1-AS1 can regulate numerous biological processes, such as cell proliferation, migration and invasion through different mechanisms. In this article, we systematically summarize the recent research progress of FEZF1-AS1 in NSCLC, which might be a novel target for clinical therapy.

近年来肺癌的发病率和死亡率均呈明显上升趋势，据统计^[1]^，2018年全球肺癌新增病例约210万，而死亡病例高达176万。根据肺癌的临床和组织病理学特征，可分为非小细胞肺癌（non-small cell lung cancer, NSCLC）和小细胞肺癌，其中NSCLC占80%-85%，又分为腺癌、鳞状细胞癌和大细胞肺癌等。NSCLC预后较差，尽管近几年分子靶向治疗和免疫疗法层出不穷，但因为许多患者在发现时已经处于疾病的晚期，5年生存率仍不足20%^[2]^，且继发性耐药的产生极大限制了其临床疗效^[3]^。因此，深入研究NSCLC发生、发展及耐药的分子机制，寻找早期诊断的生物标志物和有效的治疗靶点，对于改善患者的预后意义深远。

随着生物信息学和分子生物学技术的进步，人们对长非编码RNA（long non-coding RNA, lncRNA）的研究不断深入，越来越多的证据显示，lncRNA在表观遗传、染色体修饰、转录激活等生物学过程中作为关键调控因子，发挥着重要的作用^[4]^。FEZ家族锌指1反义RNA1（FEZ family zinc finger 1-antisense RNA 1, FEZF1-AS1）是一种新发现的lncRNA，在结肠癌、NSCLC、胰腺癌、乳腺癌等多种恶性肿瘤中表达异常，与癌症的发生发展密切相关，在肿瘤的基因诊断、治疗和预后评估等方面有着潜在的应用前景^[4-8]^。本文将结合国内外文献，将FEZF1-AS1在NSCLC中的作用机制及研究进展作一阐述，为寻找NSCLC的诊疗方法提供新思路。

## 1 LncRNA简介

在人类基因组中超过90%的基因转录为RNA，但无法编码蛋白质，被统称为非编码RNA（non-coding RNA, ncRNA）。LncRNA是一类转录本长度超过200个核苷酸的ncRNA，根据其与相邻编码基因的位置关系可以大致分为正义lncRNA、反义lncRNA、双向lncRNA、基因间lncRNA以及基因内lncRNA几类^[5]^。大量研究发现lncRNA参与了表观遗传调控、细胞周期及分化控制、染色质修饰、转录、翻译等一系列生物学过程^[4]^。LncRNA的功能与其在细胞内的定位有关，大多数核内lncRNA通过招募DNA甲基化转移酶和组蛋白修饰基因来指导染色质修饰，从而发挥转录抑制作用。此外lncRNA的自身转录也可以调控其附近位点或远处基因的表达。而细胞质中lncRNA主要通过碱基配对调节翻译过程，同时它们还可以作为竞争性内源性RNA（competing endogenous RNA, ceRNA）结合并阻断特异性的微小RNA（microRNA, miRNA），维持靶mRNA的稳定性。LncRNA可以作为肿瘤促进或抑制因子调节癌细胞的凋亡、增殖和分化能力，并且与肿瘤耐药性密切相关，使其成为当今分子生物学研究的热点^[6]^。

## 2 FEZF1-AS1在肿瘤中的作用

FEZF1-AS1最早是于2004年由日本学者在分离人类全长cDNA时发现的，它位于人基因组7号染色体7q31.32上，全长2, 653 bp，包含了7个外显子区^[7]^。FEZF1-AS1属于反义lncRNA，因其第一个外显子区和FEZF1 mRNA的第一个外显子区存在611个核苷酸的互补配对，从而命名为FEZF1-AS1^[8]^。目前已有研究表明，*FEZF1-AS1*作为癌基因在肿瘤细胞的增殖、迁移、侵袭等过程中起着十分重要的作用。

Gu所在团队^[9]^通过高通量测序及癌症基因组图谱（The Cancer Genome Atlas, TCGA）数据库分析等方法筛选出了包括FEZF1-AS1在内的9种差异表达基因，并且发现它们对胃腺癌具有潜在的诊断价值。*Kaplan-Meier*分析显示FEZF1-AS1高表达患者的总生存时间（overall survival, OS）及无复发生存时间（relapse-free survival, RFS）较短。受试者工作特征曲线（receiver operating characteristic curve, ROC）结果表明FEZF1-AS1在胃癌诊断中具有较高的灵敏度和特异性^[10]^。Liu等^[11]^分别检测了胃癌患者、胃良性病变患者及健康人血清中FEZF1-AS1的表达情况。结果表明，与胃良性病变组和对照组相比，胃癌患者FEZF1-AS1水平显著升高。同时，试者工作特征曲线分析发现FEZF1-AS1灵敏度和特异度明显高于常规肿瘤标志物癌胚抗原（carcinoembryonic antigen, CEA）和糖类抗原19-9（carbohydrate antigen19-9, CA19-9）。联合检测血清FEZF1-AS1、CEA和CA19-9可以增强胃癌诊断的敏感性。此外，他们发现胃癌术后患者血清中FEZF1-AS1水平较术前明显下降。这些结果表明循环FEZF1-AS1可作为胃癌的潜在生物标志物来进行动态监控。在机制方面，Liu等^[12]^认为FEZF1-AS1可以通过结合组蛋白去甲基化酶1（lysine specific demethylase 1, LSD1）介导p21的启动子区组蛋白第三亚基四号赖氨酸的二甲基（dimethylation of lysine 4 on histone H3 protein subunit, H3K4me2）去甲基化，在表观遗传学水平上抑制p21表达，从而促进胃癌细胞的增殖。Wu等^[10]^则发现沉默FEZF1-AS1后，β-catenin、c-myc和cyclin D1的表达降低，提示FEZF1-AS1可能通过激活Wnt/β-catenin信号通路来促进胃癌的发生和发展。

Chen等^[13]^应用qRT-PCR检测到FEZF1-AS1在结直肠癌组织及细胞系中均呈高表达。FEZF1-AS1的表达水平与T分期、淋巴结转移及远处转移密切相关。单因素和多因素分析表明FEZF1-AS1的高表达是结直肠癌的独立预后因素。他们提出FEZF1-AS1主要通过调控细胞周期来促进肿瘤细胞的增殖，且敲低FEZF1-AS1能抑制结直肠癌细胞的迁移和侵袭能力。Bian等^[14]^通过裸鼠体内成瘤实验发现FEZF1-AS1能够促进结直肠癌的肺转移及肝转移。蛋白质体外结合（RNA pull-down）实验及质谱分析结果预测丙酮酸激酶M2（pyruvate kinase 2, PKM2）是FEZF1-AS1的相关蛋白，生物学信息分析又发现转录激活因子3（signal transducers and activators of transcription 3, STAT3）可以被FEZF1-AS1和PKM2所调控。他们认为FEZF1-AS1可能通过增强PKM2的活性，促进肿瘤细胞的需氧糖酵解，激活STAT3信号通路来影响癌细胞的增殖和转移。

乳腺癌是女性中最常见的恶性肿瘤，严重影响了女性患者健康及生活质量。而FEZF1-AS1在乳腺癌中同样发挥着重要的作用，研究发现FEZF1-AS1可以降低乳腺癌干样细胞的CD44^+^/CD24^-^比率和成球能力，且敲低FEZF1-AS1可以减少Nanog、Oct4、Sox2等干细胞因子的表达。进一步的研究^[15]^表明，FEZF1-AS1通过与miR-30a结合来调节Nanog蛋白的表达，促进乳腺癌的发生发展。

## 3 FEZF1-AS1与NSCLC的预后及发生发展

He及其团队^[16]^通过qRT-PCR检测发现，与正常癌旁组织及人肺上皮细胞（16HBE）相比，FEZF1-AS1在NSCLC组织和细胞系中的表达显著上调。统计分析表明，FEZF1-AS1的表达水平与淋巴结转移、TNM分期以及肿瘤分化程度正相关。Gong等^[17]^检测了160例NSCLC组织及其邻近非肿瘤组织中FEZF1-AS1的表达水平。结果显示，FEZF1-AS1在肿瘤组织中表达显著上调，并且其高表达与更高级别的肿瘤分期和肿瘤家族史有关。刘等^[18]^则发现肺腺癌组织中FEZF1-AS1的表达水平与组织学分级和淋巴结转移相关，同时*Kaplan-Meier*曲线分析结果提示FEZF1-AS1是NSCLC的独立预后因素，FEZF1-AS1高表达的患者生存时间显著缩短。

为探索FEZF1-AS1在NSCLC中的生物学作用，He等^[16]^使用si-RNA敲低了A549及SPC-A1细胞中FEZF1-AS1的表达。MTT细胞增殖试验和克隆形成试验证实，与si-NC组相比FEZF1-AS1下调降低了肺癌细胞的细胞增殖能力。此外，Transwell实验表明敲低FEZF1-AS1的表达对肿瘤细胞的侵袭力有着显著的抑制作用。有研究^[19]^证实细胞上皮-间质转化（epithelial-mesenchymal transition, EMT）过程与恶性肿瘤的侵袭和转移密切相关。EMT是指上皮细胞丧失细胞极性以及细胞间的黏附连接能力，转化成为具有间质表型的细胞，同时EMT还伴随着关键性上皮性标志物E-钙黏蛋白（E-cadherin）以及间质性标志物波形蛋白（Vimentin）的改变。在Western blot试验中，他们发现FEZF1-AS1可以降低NSCLC细胞中E-cadherin及ZO-1蛋白表达，并增加Slug、Twist和Vimentin的表达水平。而E-cadherin是细胞侵袭和EMT过程中的关键调控因子，表明FEZF1-AS1可以通过抑制EMT促进NSCLC细胞的增殖和侵袭。目前研究普遍认为lncRNA可以通过与RNA结合蛋白作用来调控潜在的靶标。随后He团队进行了RNA结合蛋白免疫沉淀（RNA binding protein immunoprecipitation, RIP）和染色质免疫沉淀（chromatin immunoprecipitation assay, ChIP）实验，结果证实FEZF1-AS1可以直接结合Zeste基因增强子同源物2（enhancer of zeste homolog 2, EZH2）和LSD1，而敲低FEZF1-AS1则会降低EZH2及LSD1与E-cadherin启动子区域的结合能力，即沉默FEZF1-AS1可以上调E-cadherin的表达。此外，研究还发现FEZF1-AS1表达下调后可以增加A549细胞中Axin1的表达，并降低β-catenin和TCF4的表达，这表明FEZF1-AS1也可以通过调节NSCLC中的Wnt/β-catenin信号传导活性来参与EMT进程。

Jin等^[20]^发现沉默FEZF1-AS1可将肺腺癌细胞阻滞在G_1_期，从而抑制肺癌细胞增殖、促进其凋亡。进一步的研究^[21]^表明，在肺癌细胞中敲低FEZF1-AS1后p57的表达升高。*P57*因其对于肿瘤细胞增殖起负性调节作用，而被认为是种抑癌基因。目前已有研究发现，人肺癌中存在着*p57*基因组印记的缺失，p57可以通过抑制细胞周期蛋白依赖激酶以及增殖细胞核抗原，实现对细胞周期的调控。核质分离实验结果显示，FEZF1-AS1在细胞核中的表达明显高于细胞质，提示FEZF1-AS1可能主要在转录水平发挥作用。随后的RIP实验及RNA pull-down实验证实，FEZF1-AS1能够与RNA结合蛋白EZH2和LSD1直接绑定。ChIP测定发现FEZF1-AS1主要通过将EZH2及LSD1募集到p57的启动子区域，并诱导组蛋白修饰来调节肺腺癌细胞中的p57表达，从而促进肺癌的发生和发展。

有研究认为反义RNA可以通过直接与互补的mRNA序列结合在转录后水平调节亲本编码基因的表达。Gong等^[17]^为探究FEZF1-AS1作为反义RNA是否也存在类似作用机制，检测了58例NSCLC组织中FEZF1-AS1及FEZF1的表达水平，发现FEZF1-AS1的表达水平和FEZF1呈正相关。随后研究者又通过GEPIA数据库分析了969例NSCLC患者中FEZF1-AS1和FEZF1的表达，并得到了相似的结论。同时他们还发现两者的外显子存在部分重叠，猜测FEZF1-AS1可能通过结合*FEZF1*基因调节其表达。在胃癌中，有研究指出FEZF1-AS1的启动子区包含一个保守的SP1结合位点，而*FEZF1*基因启动子区去甲基化，组蛋白乙酰化和转录因子SP1结合可诱导*FEZF1*基因转录激活，而肺癌中相关的作用机制还有待进一步探讨^[12]^。刘等^[18]^发现在肺癌细胞中敲低FEZF1-AS1后其有义同源基因FEZF1 mRNA和蛋白的表达均受到抑制。而使用小干扰RNA下调*FEZF1*基因的表达后FEZF1-AS1的表达水平也明显下降。为了证明FEZF1-AS1和FEZF1之间的相互作用，研究者在肺癌细胞系中进行了双荧光素酶报告基因检测，结果表明干扰FEZF1-AS1表达无法改变FEZF1的荧光素酶活性。

## 4 FEZF1-AS1与EGFR-TKIs耐药相关的可能机制

肺癌治疗是目前的研究热点，因传统放化疗临床疗效有限，患者5年生存率不足15%。以表皮生长因子受体酪氨酸激酶抑制剂（epidermal growth factor receptor-tyrosine kinase inhibitors, EGFR-TKIs）为代表的分子靶向治疗的出现是肺癌治疗领域突破性的进展，大幅延长了患者的生存期，提高了患者生存质量。在我国约有50%的肺腺癌患者携带有*EGFR*基因突变，除部分原发耐药患者外（约10%），EGFR-TKIs对于*EGFR*敏感突变的患者疗效显著。然而随之而来的获得性耐药的产生，成为了临床抗肿瘤治疗的一大难题。常见的耐药机制包括*EGFR* T790M、*c-MET*扩增、*HER2*扩增、*BRAF*突变及小细胞肺癌转化等，但有约30%的机制仍不明确。有研究^[22]^显示lncRNA在耐药细胞及组织中表达异常，为EGFR-TKIs的耐药机制研究提供了新的思路。例如lncRNA UCA1、MIR31HG可以通过激活PI3K/AKT信号通路诱导NSCLC细胞对吉非替尼产生耐药性^[23, 24]^。BC087858可能通过调控EMT进程在EGFR-TKIs获得性耐药中发挥作用^[25]^，而FEZF1-AS1也被证实是这些通路中的重要调节因子。Li等^[26]^发现miR-610能够与FEZF1-AS1的3’端非翻译区（3-untranslated region, 3’UTR）结合，且AKT3的表达与miR-610的表达呈负相关。沉默FEZF1-AS1可以降低AKT3 mRNA和蛋白的表达，而FEZF1-AS1与AKT3的表达呈正相关，表明FEZF1-AS1可以通过调节miR-610/AKT3轴以促进多发性骨髓瘤细胞的增殖。Wang等^[27]^提出JAK2/STAT3信号通路在肝癌细胞EMT进程中起着不可或缺的作用，沉默FEZF1-AS1能够抑制STAT3的磷酸化，且敲低FEZF1-AS1后N-钙粘蛋白（N-cadherin）和Vimentin表达降低，E-cadherin表达升高，从而抑制了EMT过程。然而，FEZF1-AS1在EGFR-TKIs耐药中的作用机制仍需进一步深入研究，为实现其临床潜力提供新的理论基础。

## 5 总结及展望

综上所述，lncRNA *FEZF1*-*AS1*作为一种致癌基因，通过招募多梳抑制复合体、竞争内源性RNA、调控信号通路等方式在NSCLC细胞增殖、侵袭、转移等恶性生物学过程中发挥关键的调节作用。全面深入地了解FEZF1-AS1，也将在NSCLC的诊断、治疗以及预后评估等临床应用中产生重要的价值。
